# Advances in Biomarkers for PCa Diagnostics and Prognostics—A Way towards Personalized Medicine

**DOI:** 10.3390/ijms18102193

**Published:** 2017-10-20

**Authors:** Carsten Stephan, Klaus Jung

**Affiliations:** 1 Department of Urology, Charité University Hospital, 10117 Berlin, Germany; klaus.jung@charite.de; 2 Berlin Institute for Urologic Research, 10115 Berlin, Germany

## 1. Introduction

Prostate cancer (PCa) is, with an estimated number of 161,360 cases and 26,730 deaths in 2017, the most common malignancy in the USA [1]. Worldwide, the mortality rates tend to be higher in less developed regions, including parts of South America, the Caribbean, and Africa [2]. The overall PCa incidence in all countries with available data for 2008 was counted with almost 900,000 cases while the mortality rate was estimated with approximately 258,000 deaths from PCa [2]. Thus, worldwide almost 30% of men with PCa will die of this malignancy.

In recent years, many new developments in PCa diagnostics were reviewed. Numerous new protein- and nucleic acid-based biomarkers in whole blood and its fractions serum or plasma as well in urine and its different fractions were described based on novel technologies [3,4,5,6,7]. To reduce overdiagnosis and overtreatment there is a clear focus to preferentially detect clinical significant PCa because indolent PCa often do not need treatment or treatment can be delayed. Here a multimodal risk score was developed in a study on urine samples with two independent prospective multicenter cohorts [8]. The multimodal approach including the *HOXC6* and *DLX1* mRNA levels reached excellent area under the Receiver-Operating-Characteristic (ROC) curve (AUCs) of 0.90 and 0.86 in the training and validation cohort [8]. Beside the diagnostics, also the risk stratification [9] and prognostic factors [10] are important for the progress of the disease.

The treatment options with several newly available substrates for advanced PCa, including castration-resistant prostate cancer (CRCP), have been changed dramatically within recent years, but no biomarker has been validated so far [11]. The androgen-receptor splice variant 7 (AR-V7) protein expression and its localization in circulating tumor cells (CTCs) should also be highlighted [12], but there is a need for further prospective studies to validate this biomarker. As an example, the measurement of epidermal growth factor receptor (EGFR) on CTCs might be promising as a prognostic marker in metastatic PCa patients [13].

## 2. Overview

This Special Issue contains 26 papers consisting of 22 original research studies and four reviews. Five studies present their basic research data using PCa cell lines [14,15,16,17] or summarize data on cancer/testis antigens (CTAs) as potential PCa biomarkers [18]. Serum markers are the subject in the vast majority of publications (*n* = 8) with focus on PCa diagnostic and prognostic [19,20,21,22,23] or evaluation of advanced/metastatic disease stages [24,25,26]. Two of the four reviews further summarize biomarkers for PCa diagnostic [27] or active surveillance [28] while the other two reviews have special topics like nanoparticles as theranostic vehicles [29] or PCa stem cells [30]. Four working groups published results by using immunohistochemistry in prostate, lymph node or bone tissue [31,32,33,34]. This Special Issue also covered studies on seminal plasma biomarkers [35], improved PET contrast between osteolytic and osteoblastic bone metastatic lesions with fasting [36] and the impact of the blood collection tube and storage time on CTC-mRNA (AR-V7) analysis [37]. Two clinical studies on different biomarkers for prostate brachytherapy [38,39] complete the large number of publications.

## 3. Basic Research

Metformin, known as an anti-diabetes drug, has been shown to have anti-neoplastic effects in several tumors including PCa. Metformin targets Hedgehog (Hh) signaling, which is an important target for radiosensitization. Gonnissen et al. [14] evaluated the combination of metformin and the Hh inhibitor GANT61 with or without ionizing radiation in the three PCa cell lines PC3, DU145, and 22Rv1. Although this drug combination reduced the cell growth and enhanced the radiosensitization effect compared to single agent in cell lines, both in vitro effects could not be confirmed in vivo [14]. This observation shows the limitation of in vitro testing and the importance of careful translation of successful in vitro experiments under in vivo setting conditions.

Dayal et al. [15] showed that mutations in RNase L may promote PCa by increasing expression of androgen receptor (AR)-responsive genes and cell motility and they identified novel roles of RNase L as a PCa susceptibility gene. For instance, the activity of the two matrix metalloproteinases (MMP)-2 and -9 is significantly increased in cells where RNase L levels are ablated [15]. The imbalance between MMP-9 activity and its inhibitory counterparts in prostate cancerous tissue already implicated a rationale of using synthetic inhibitors of MMPs as potential therapeutic tools [40] that was also confirmed in a prostate cancer standard rat model [41].

In advanced PCa, small ubiquitin-like modifier (SUMO)-specific cysteine protease 1 (SENP1) is up-regulated. In their study, Zhang et al. [16] developed a lentiviral vector, to silence SENP1 in prostate cancer cells with high metastatic characteristics (PC3M). The researcher further created an adenovirus vector to over-express SENP1 in prostate cancer cells with low metastatic potential (LNCaP). The authors could show that silencing of SENP1 promoted cellular apoptosis and they concluded finally that SENP1 is a potential target for treatment of advanced PCa [16]. Further studies including first clinical trials are necessary.

Bascetta et al. [17] isolated PCa cells resistant to docetaxel (DCTR) clones from different PCa cell lines and performed through next-generation sequencing of the released miRNAs. They identified several miRNAs, which were differentially released in the growth medium [17]. The authors proposed that the utilization of clones resistant to a given drug as in vitro model to identify the differentially released miRNAs, could be tested in perspective as predictive biomarkers of drug resistance in tumor patients under therapy [17]. This is an interesting theory. However, measurements of these miRNAs in plasma/serum must follow to find evidence of this approach.

As a basic step for numerous experiments, Australian researchers determined the ideal blood storage conditions to preserve CTC-specific mRNA biomarkers [37]. Luk et al. [37] tested the preservation of tumor cells and CTC-mRNA over the time in ethylene-diamine-tetra-acetic acid (EDTA) and acid citrate dextrose solution B (Citrate) blood collection tubes in comparison to special cell-free DNA, RNA and Cyto-Chex blood collection tubes (Streck, Omaha, NE, USA). Tumor mRNA biomarkers were readily detectable after 48 h storage in conventional EDTA and Citrate tubes, but not in the three specially developed preservative-containing collection tubes. Notably, AR-V7 expression was detected in PCa patient blood samples after 48 h storage in EDTA tubes at room temperature, leaving a more feasible timeframe compared to previous recommendations [37].

The cancer/testis antigens (CTAs) are a group of proteins that are typically restricted to the testis in the normal adult but are aberrantly expressed in several other types of cancers including PCa. Using prostate-associated gene 4 (PAGE4) as an example of a disordered CTA, Kulkami and Uversky [18] highlighted how intrinsically disordered proteins (IDP) conformational dynamics may regulate phenotypic heterogeneity in PCa cells, and how it may be exploited both as a potential biomarker as well as a promising therapeutic target in PCa. The authors favor the theranostic potential of CTAs that is latent in their lack of structure and casts them as next generation or “smart” biomarker candidates [18]. This idea should be supported but the markers should be disease specific.

## 4. Serum Biomarkers

Hagiwara and colleagues [19] evaluated the performance of Wisteria floribunda agglutinin (WFA)-reactive glycan-carrying PSA-glycosylation isomer (PSA-Gi) in serum by using an automated immunoassay system in 244 patients with PCa and 184 with biopsy-proven benign prostate hyperplasia (BPH). The area under the ROC curve (AUC) for PSA-Gi was almost 0.8, which was higher than for PSA (0.64) or the PSA-Gi/PSA ratio (AUC 0.73). The correlation between PSA-Gi and the Gleason grade is a further very positive aspect that implies that PSA-Gi is a promising marker not only for detecting PCa but also for assessing its aggressiveness [19]. A tissue-based nomogram was also developed as a predictive tool for determining the PSA-free survival probability [19].

Another research group from Japan worked on the PCa-associated α2,3-linked sialyl N-glycan-carrying PSA (S2,3PSA) [20]. The authors estimated PSA and S2,3PSA in each of 50 age and PSA matched biopsy-proven patients with PCa and BPH (from a larger cohort of >550 biopsied men) by a newly developed automated micro-total immunoassay system with a detection limit of 0.05 ng/mL [20]. The authors can be congratulated for the development of a robust and reproducible immunoassay with a coefficient of variation within 15% that is superior to their earlier magnetic bead-based S2,3PSA assay [42]. The AUC for the S2,3PSA to PSA ratio (%S2,3PSA) was 0.834 and much superior to PSA alone (AUC: 0.506) [20]. However, a comparison of %S2,3PSA with the FDA-approved and currently best available PSA-based serum marker prostate health index (PHI; formula: −2proPSA/free PSA × √PSA) has not been performed. It would be very interesting if %S2,3PSA could further improve PHI in future prospective multicenter studies.

An independent research group also published data with their 2015 patented PSA glycoform assay [43], based on the determination of the α2,3-sialic acid percentage of serum PSA (%α2,3-SA). The current study compared PHI with %α2,3-SA in a cohort of 79 patients that included 50 PCa and 29 with BPH [22]. The %α2,3-SA could distinguish high-risk PCa patients from the rest of patients better than PHI (AUC 0.971 vs. 0.840), although PHI correlated better with the Gleason score than the %α2,3-SA [22]. The combination of both markers increased the AUC up to 0.985 resulting in 100% sensitivity and 94.7% specificity to differentiate high-risk PCa from the other low and intermediate-risk PCa and BPH patients [22]. The editor is eagerly awaiting multicenter data on the new %α2,3-SA assay. It would be further interesting to compare both α2,3-sialic acid assays from Ishikawa et al. [20] and Ferrer-Batalle et al. [22] in view of its introduction as soon as possible in clinical practice.

Friedersdorff et al. [21] investigated the relationship between PHI, Gleason Score and prostate tumor volume in almost 200 prostatectomy specimen. With an AUC of 0.79, PHI was the most accurate predictor of a tumor volume > 0.5 cm^3^ [21]. Most important, PHI correlated significantly with the tumor volume (*r* = 0.588), which is significantly better (*p* = 0.008) than the correlation of the Gleason score with tumor volume (*r* = 0.385). This shows that the gold standard of Gleason Score has been surpassed regarding its value on tumor size. Further, our own data regarding PCa prognosis also show the value of PHI with an improved prediction of biochemical recurrence (BCR) [44]. PHI obviously outperforms other diagnostic PCa biomarkers (urinary PCA3, TMPRSS2:ERG) with its good association to tumor aggressiveness, tumor volume and prognosis [45,46,47].

PHI was again the topic of a study by Schlack and colleagues [25]. In 25 patients with metastatic castration resistant prostate cancer (mCRPC), following initiation of Abiraterone-therapy, the PSA-subforms were analyzed before and at 8–12 weeks under therapy as prognosticators of progression-free-survival (PFS) and overall survival (OS). Comparing patients with a PFS < vs. ≥ 12, the relative-median-change of PSA, free PSA and −2proPSA differed significantly [25]. Decreasing free PSA and −2proPSA values indicated an OS of 32 months compared with 21 months in men with rising values [25]. Univariate and multivariate Cox regression analyses could not prove all these tests as suitable predictive PFS and OS markers [25]. However, the authors stressed the limitation of their study due to the small sample size in a single center.

In the last diagnostic study with serum, high throughput sequencing of small RNAs extracted from blood from 28 untreated PCa patients and 12 healthy controls was used to identify microRNAs as PCa biomarker [23]. Four microRNAs (miR-127-3p, miR-204-5p, miR-329-3p and miR-487b-3p) were upregulated, three miRNAs (miR-32-5p, miR-20a-5p and miR-454-3p) were downregulated. ROC curves exhibited a better correlation with PCa than for PSA [23]. It should be emphasized that the selection of four miRNA as normalization standard is a very positive additional part of this study. However, the AUCs for the single miRNAs are between 0.75 and 0.95, but the number of patients is quite low and comparing not only with PSA but also at least %free PSA or better PHI would improve this comparison. In addition, some of the suggested miRNAs (miR-20a-5p, miR-32-5p, and miR-454-3p) are hemolysis-affected [48]. The use of such hemolysis-affected circulating miRNAs has been recently questioned without a special control of hemolysis [49].

Geng et al. [24] checked first whether genetic variants in the Wnt pathway influence clinical outcomes for advanced PCa patients receiving androgen deprivation therapy (ADT). In 465 PCa patients, two common single nucleotide polymorphisms (SNPs), the adenomatous polyposis coli (APC) rs2707765 and rs497844, were significantly associated with both PCa progression and all-cause mortality [24]. This may implement a preclinical rationale for using APC as a prognostic marker in advanced PCa by identifying patients who would not benefit from ADT [24].

In 96 patients with mCRPC under a median time of 10 months with Abiraterone treatment a serum pre-treatment neutrophil-to lymphocyte-ratio (NLR) < 5 was associated with better survival outcomes [26]. Contrary, the authors found that after eight weeks of Abiraterone therapy, a change of initially elevated NLR of >5 to <5 was associated with worse survival. Therefore, a deeper understanding of the underlying immune mechanisms in this setting is highly warranted [26].

## 5. Reviews

Filella and Foj [27] extensively reviewed all important biomarkers for early detection of PCa, also regarding the important point of overdetection and false positive results. This excellent compilation provides an overview of all PSA subforms and the timeline of PCa biomarkers since 1970 [27]. Beside the emerging role of PHI, also the use of the urine based markers PCA3 (FDA approved in 2012 for men older than 50 who have at least one previous negative biopsy) and TMPRSS2:ERG fusion gene is provided. Furthermore, aberrant microRNA and exosomal biomarkers are reviewed [27].

Ferro and colleagues [28] provided a comprehensive overview of biomarkers as predictors of clinical significant PCa and for PCa patients under active surveillance. The main topics were further epigenetic signatures with DNA methylation, histone modifications, and noncoding RNA, which all could potentially provide new tools for PCa prognosis [28].

Individualized targeted theranostic nanomedicine has emerged by using nanoparticles as vehicles carrying both diagnostic and therapeutic molecular entities. Nanomedicine can increase sensitivity and specificity on diagnosis and might be used for improved survival or prolonged survival after therapy [29]. A large review by Dr. Elgqvist [29] presents and discusses important and promising different kinds of nanoparticles, as well as imaging and therapy options, suitable for theranostic applications. Beside breast cancer, PCa is presented in detail regarding diagnosis, staging, recurrence, metastases, and treatment options that are available today, followed by possible ways to move forward applying theranostics. This very comprehensive review encompasses 53 pages and almost 600 references [29].

The hot topic of the last of the four reviews by Zhang et al. [30] included cancer stem cells biomarkers including a few novel markers discovered recently. Those biomarkers might play an important role to detect resistance to traditional cancer therapies [30]. Further studies of cancer stem cells (including specific isolation and targeting on those cells) might be helpful for the discovery of novel treatments for prostate cancer, especially castration resistant disease [30].

## 6. Immunohistochemistry and Other Methods

In the first accepted paper of this Special issue by Chen et al. [31] the authors used tissue microarray and immunohistochemistry to estimate the phosphorylated levels of Akt (p-Akt) in 53 radical prostatectomy (RP) specimen. Within a Cox proportional hazard model a high p-Akt image score better predicted (hazard ratio (HR): 3.12) the risk of BCR than a high Gleason score (HR: 1.18) or a high PSA (HR: 0.62, *p* = 0.57) [31]. It should be noted that the initial 76 RP specimen (23 had to be excluded) were collected over a relatively long time from 1999 to 2011 [31]. A limitation of this second study from Taiwan was a much higher mean PSA (almost 30 ng/mL) in the group of high p-Akt Scores ≥ 8 as compared with the group with a p-Akt Score ≤ 6, where the mean PSA was only 12 ng/mL. Therefore, the conclusion that p-Akt activation can potentially determine BCR in pT2 PCa patients after RP should be taken with caution. Regarding BCR after RP, a very recent publication of our own working group by Zhao et al. [50] comprehensively reviews biomarkers with a special focus on miRNAs and its combinations to improve PCa prognosis. There is obviously a theranostic utility and a diagnostic, prognostic and future therapeutic potential of miRNAs in prostate cancer [51].

Campos et al. [32] investigated in a pilot study the expression of the epithelial cell adhesion molecule (EpCAM) in PCa lymph nodes and matched normal lymph nodes, in PCa bone metastases, and in normal bone by immunohistochemistry. EpCAM was expressed in 100% of lymph node metastases (*n* = 21), in 0% of normal lymph nodes (0 out of 21), in 95% of bone metastases (19 out of 20), and in 0% of normal bone (0 out of 14) [32]. Based on these results, EpCAM may be a feasible imaging target in PCa lymph node and bone metastases [32]. If prospective trials can confirm these promising results, EpCAM may help to improve pretherapeutical staging and to detect possible micro metastasis.

Another survey by Genitsch et al. [33] also tested the neuroendocrine differentiation (NED) by Chromogranin A expression in lymph node metastases, as well as primary tumors, from 119 consecutive PCa patients. The mean percentage of NED cells increased significantly (*p* < 0.001) from normal prostate glands (0.4%), to primary prostate cancer (1.0%) and nodal metastases (2.6%). However, in primary tumors and nodal metastases, tumor areas with higher Gleason patterns tended to display a higher NED, but no significance was reached [33].

A third study in lymph node metastases evaluated the homeobox protein Hox-B13 (HOXB13), which has been suggested as a new marker for the detection of prostatic origin [52]. Kristiansen et al. [34] semi-quantitatively compared the diagnostic value of different immunohistochemically markers such as PSA, Prostatic acid phosphatase (PSAP), prostate specific membrane antigen (PSMA), homeobox gene *NKX3.1*, prostein, androgen receptor (AR), HOXB13, and the ETS-related gene (ERG) in 64 lymph node metastasis. The detection rate of prostate origin of metastasis for single markers was 100% for NKX3.1, 98.1% for AR, 84.3% for PSMA, 80.8% for PSA, 66% for PSAP, 60.4% for HOXB13, 59.6% for prostein, and 50.0% for ERG [34]. Thus, HOXB13 alone lacks sensitivity for the detection of prostatic origin, so the combination of PSA and NKX3.1 should be preferred.

Based on their previous work on PCa detection with naturally occurring fragments of the larger seminal proteins semenogelin 1 and 2 in seminal plasma by using CE-MS/MS [53], the group of Neuhaus et al. [35] identified proteases putatively involved in PCa specific protein cleavage, and further examined gene expression and tissue protein levels. They found different MMP3 and MMP7 activity in PCa compared with BPH due to fine regulation by their tissue inhibitor TIMP1 [35]. These data support the old idea of non-invasive seminal plasma biomarkers as additional tool for PCa detection and risk stratification. However, tests in seminal plasma seem to be of little acceptance in clinical practice.

A third Japanese research group investigated the influence of fasting on fluciclovine-PET using a triple-tracer autoradiography in a rat breast cancer model of mixed osteolytic/osteoblastic bone metastases in which the animals fasted overnight [36]. Their in vivo and in vitro results suggest that fasting before 18F-fluciclovine-PET improves the contrast between osteolytic and osteoblastic bone metastatic lesions and background, which might facilitate a clearer visualization of lesions in fluciclovine-PET imaging [36].

## 7. Brachytherapy

The last two studies of this Special issue present data on patients receiving prostate brachytherapy [38,39]. In the sixth study from Germany by PD Ecke et al. [38] PSA, PSA density and other clinical data were evaluated before and after brachytherapy with external beam radiation in 79 high-risk PCa patients treated between 2009 and 2016. PSA density and PSA at time of diagnosis (*p* = 0.009 and 0.033), and PSA on date of first follow-up after one year (*p* = 0.025) were significant predictors for local recurrence during follow-up [38]. The authors further concluded that their specific radiation therapy for high-risk PCa resulted in high biochemical control rates with minimal side-effects [38].

In the fourth contribution from Japan, Tsumura et al. [39] measured CTCs before and during brachytherapy in 30 high-risk and 29 low-risk PCa patients. While no preoperative sample showed CTCs (0%), they were detected in intraoperative samples in 7 of the 59 patients [39]. The authors could not find any association of intraoperative CTC increases with clinicopathological data at all [39]. This may reflect the fact that all patients undergoing brachytherapy have a certain risk of intraoperative haematogenous spillage of prostate cancer cells, irrespective of use of neoadjuvant hormonal therapy, type of brachytherapy, PSA and other clinical data. This lowers a possible impact of CTCs in the diagnosis or prognosis of PCa. Further, there is also the possibility that normal epithelial cells are transiently released into the blood stream due to brachytherapy procedures and therefore additional tests would need to confirm if cells released in this manner are indeed real CTCs.

## 8. Summary

In summary, this Special Issue of the International Journal of Molecular Sciences on diagnostic, prognostic and predictive biomarkers in PCa is an excellent compilation of 26 publications accepted between July 2016 and July 2017 with authors from 15 different countries.

As recently reviewed by Ali et al. [54], serum, urine, tissue and imaging biomarkers have been widely evaluated to improve the identification of clinically significant PCa. Importantly, changes in MRI technology such as multiparametric MRI (mpMRI) have realized a quantum change, and this facility is now becoming more widely incorporated into diagnostic and disease risk-stratification protocols [54]. An example on combining biomarker and mpMRI results has been published very recently (October 2017) by Hendriks et al. [55]. The researchers determined the association between a urinary biomarker-based risk score (SelectMDx) and the mpMRI based prostate imaging reporting and data system (PI-RADS) score regarding prostate biopsy results [55]. With an AUC of 0.83 for SelectMDx (compared to 0.66 for PSA and 0.65 for PCA3) it was further not surprising that there was a positive association between the SelectMDx score and the PI-RADS score with significant differences between PI-RADS 3 and 4 (*p* < 0.01) and between PI-RADS 4 and 5 (*p* < 0.01) in the SelectMDx score [55]. Analogous to the older concept of PSA density (PSA divided by prostate volume) the new term of PHI density (PHI/prostate volume) was described in 2017 as another marker combination by Tosoian et al. [56].

Beside these new developments in urinary markers [5,8,55] and the PHI density [56], the serum immunoassays with the PCa-associated aberrant glycosylation of PSA (S2,3PSA) published here should be highlighted [20,22].

To conclude, the identification of clinically significant PCa by biomarkers and image modalities (mpMRI) is a step towards personalized diagnosis, which will be the future. Also for advanced PCa, results of this Special issue [24] might influence personalized PCa management.
